# The COMPARE Database: A Public Resource for Allergen Identification, Adapted for Continuous Improvement

**DOI:** 10.3389/falgy.2021.700533

**Published:** 2021-08-06

**Authors:** Ronald van Ree, Dexter Sapiter Ballerda, M. Cecilia Berin, Laurent Beuf, Alexander Chang, Gabriele Gadermaier, Paul A. Guevera, Karin Hoffmann-Sommergruber, Emir Islamovic, Liisa Koski, John Kough, Gregory S. Ladics, Scott McClain, Kyle A. McKillop, Shermaine Mitchell-Ryan, Clare A. Narrod, Lucilia Pereira Mouriès, Syril Pettit, Lars K. Poulsen, Andre Silvanovich, Ping Song, Suzanne S. Teuber, Christal Bowman

**Affiliations:** ^1^Departments of Experimental Immunology and of Otorhinolaryngology, Amsterdam University Medical Centers, Amsterdam, Netherlands; ^2^Joint Institute for Food Safety and Applied Nutrition (JIFSAN), University of Maryland, College Park, MD, United States; ^3^Department of Pediatrics, Icahn School of Medicine at Mount Sinai, New York, NY, United States; ^4^Limagrain Field Seeds, Centre de Recherche, Route d'Ennezat, Chappes, France; ^5^Department of Biosciences, Paris Lodron University of Salzburg, Salzburg, Austria; ^6^Department of Pathophysiology and Allergy Research, Medical University of Vienna, Vienna, Austria; ^7^Regulatory Science Seeds and Traits, BASF Corporation, Morrisville, NC, United States; ^8^Health and Environmental Sciences Institute (HESI), Washington, DC, United States; ^9^Office of Pesticide Programs, Microbial Pesticides Branch, US Environmental Protection Agency, Washington, DC, United States; ^10^IFF Inc., Wilmington, DE, United States; ^11^Syngenta Crop Protection LLC, Research Triangle Park, NC, United States; ^12^Allergy Clinic, Department of Dermatology and Allergy, Gentofte Hospital, University of Copenhagen, Copenhagen, Denmark; ^13^Bayer U.S., Crop Science Regulatory Science Building FF4, Chesterfield, MO, United States; ^14^Seeds Regulatory Science, Corteva Agriscience LLC, Indianapolis, IN, United States; ^15^Department of Internal Medicine, School of Medicine, University of California, Davis, Davis, CA, United States; ^16^Division of Rheumatology, Allergy, and Clinical Immunology, Davis, CA, United States; ^17^Veterans Affairs Northern California Healthcare System, Mather, CA, United States; ^18^Formerly: Human Safety Regulatory Toxicology, Bayer CropScience LP, Research Triangle Park, NC, United States

**Keywords:** allergen database, allergenicity assessment, bioinformatics, GMO, sequence comparison, risk assessment

## Abstract

**Motivation:** The availability of databases identifying allergenic proteins via a transparent and consensus-based scientific approach is of prime importance to support the safety review of genetically-modified foods and feeds, and public safety in general. Over recent years, screening for potential new allergens sequences has become more complex due to the exponential increase of genomic sequence information. To address these challenges, an international collaborative scientific group coordinated by the Health and Environmental Sciences Institute (HESI), was tasked to develop a contemporary, adaptable, high-throughput process to build the *COM*prehensive *P*rotein *A*llergen *RE*source (COMPARE) database, a publicly accessible allergen sequence data resource along with bioinformatics analytical tools following guidelines of FAO/WHO and CODEX Alimentarius Commission.

**Results:** The COMPARE process is novel in that it involves the identification of candidate sequences via automated keyword-based sorting algorithm and manual curation of the annotated sequence entries retrieved from public protein sequence databases on a yearly basis; its process is meant for continuous improvement, with updates being transparently documented with each version; as a complementary approach, a yearly key-word based search of literature databases is added to identify new allergen sequences that were not (yet) submitted to protein databases; in addition, comments from the independent peer-review panel are posted on the website to increase transparency of decision making; finally, sequence comparison capabilities associated with the COMPARE database was developed to evaluate the potential allergenicity of proteins, based on internationally recognized guidelines, FAO/WHO and CODEX Alimentarius Commission

## Introduction

Food allergy is a growing food safety and public health concern. Well-curated, publicly accessible allergen databases serve a range of public health roles, including informing experimental research programs, clinicians, and allergists and/or providing critical data to patients or the public. In the food safety space, an allergen database serves also the purpose of evaluating concerns over the transfer of known allergens or potentially cross-reactive proteins to genetically-modified (GM) food crops or animals. In fact, regulatory agencies evaluating the safety of biotechnology products for genetically-modified food and feeds require an assessment for potential allergenicity, including comparing novel or newly discovered proteins at the level of their primary amino acid sequence to known allergens, which further emphasizes the need for a comprehensive allergen database. The goal in that assessment is to identify proteins that may be known allergens or have sufficient structural similarity to warrant further experimental investigation into their potential to elicit allergic responses based on cross-reactivity.

Therefore, allergen databases support allergy science and safety by two distinct but related processes: (1) comparative processes to identify potential similarity between query sequences and allergens using bioinformatics tools, and (2) identification of the source organism for any listed allergen, allowing researchers to assess taxonomic relatedness among the organisms producing the allergens/proteins.

Some individual companies maintain their own allergen databases. Additionally, several public databases are now available, such as the University of Texas Medical Branch's Structural Database of Allergen Proteins—SDAP (https://fermi.utmb.edu/) (1, 2), World Health Organization (WHO)/International Union of Immunological Societies (IUIS) Allergen Nomenclature Database (http://www.allergen.org/) (3–7), and AllergenOnline (http://www.allergenonline.org) supported by the University of Nebraska Food Allergy Research and Resource Program (FARRP) (8). Others include AllFam (9), Allergome (10, 11) and Allermatch (12), released in 2003 and 2004, respectively. Recently, another allergen database, AllerBase was established (13). Some additional allergen databases are available but not all are maintained regularly and have varying inclusion criteria (14). Reviews of these various allergen databases are available (15–17), in which the features and specific purposes of each resource are described. SDAP for example, can be used to find sequence and structural neighbors for an allergen, and to search for the presence of an epitope (18–20). AllerML (21) is a markup language designed to facilitate exchange of information between databases. Currently SDAP is the only allergen database using AllerML and therefore we have not yet implemented this attribute in the COMPARE database. We do not see this as an impediment to efficient searching in COMPARE as the entities in the COMPARE database are clearly annotated and distinct from the content. Data is easily available for download in both tabular and fasta formats.

COMPARE data is presently being used by other recent databases and tools: AllerCatPro, developed to predict the allergenic potential of proteins based on their 3D protein structure as well as their amino acid sequence (22) and a Random Forest allergenicity prediction model (23).

The initial number of sequences to be filtered when screening new potential allergens has grown exponentially, necessitating higher-density data handling methods to sort and identify information relevant for food safety. As the publication of genomic sequence information increased, the challenge of updating an accurate allergen database intensified as public repositories become diluted with automatically annotated sequence information and sequences that are not expressed proteins. For example, only a minute proportion of proteins in the National Center for Biotechnology Information (NCBI) Protein sequence database have been identified as allergens, with almost 60% of these belonging to four protein families (24, 25). In contrast, the February 2021 release of UniProtKB/TrEMBL counted over 200 million protein sequences; of these, over 67% are “predicted” sequences, 31.7% are inferred from homology, and only about 1% have evidence at the transcript level (0.65%) or protein level (0.08%) data obtained from “UniProtKB/TrEMBL Protein Database Release Statistics,” https://www.ebi.ac.uk/uniprot/TrEMBLstats, last accessed February 12, 2021.

To address both the dilution effect and annotation quality issues, the Health and Environmental Sciences Institute (HESI) Protein Allergens, Toxins and Bioinformatics (PATB) committee has developed the *COM*prehensive *P*rotein *A*llergen *RE*source (COMPARE) database, a new protein allergen database along with a sequence and literature screening process for public access. COMPARE's structure and process is designed to facilitate efficient annual updates. In its initial build, COMPARE started from the foundational sequence list developed by FARRP AllergenOnline database v.16, which shares COMPARE's commitment to blinded peer review and annual curation and updating, to which new sequences identified through the COMPARE process have been added to in 2017 and on each subsequent annual update.

The critical methodological components of the COMPARE database consist of scientific, structural, procedural, and quality/rigor assurance components. The COMPARE process engages a broad international multi-sector scientific steering committee with representation from government (US Environmental Protection Agency and US Food and Drug Administration [FDA]), academia, and private-sector scientists who bring real-world know-how and experience of gaps and challenges to be considered. Oversight and coordination by HESI, an independent and objective institute dedicated to advancing the understanding of scientific issues related to human health and the environment, as well as programmatic support from the Joint Institute for Food Safety and Nutrition (established between the FDA and the University of Maryland, JIFSAN; www.jifsan.umd.edu) in the development of bioinformatics analytical tools associated with the COMPARE database provide organizational support for a structured collaborative effort, made possible with pooled financial support from private sector partners, as well as significant in-kind support from both public and private sector collaborators. The quality and scientific integrity of the COMPARE resource is safeguarded through various mechanisms inherent to HESI's operating model: maintaining public-private representation, transparent documentation, and open access to the database. All database design and search algorithm decisions are publicly documented on the COMPARE website (http://www.comparedatabase.org), under the “Process Development” tab and presently, in this article.

## System and Methods

### The Database Build Process: Criteria-Based, Transparent, and Reproducible

The COMPARE process implements a consistent and transparent mechanism to identify protein allergens via the following:

1. The development of a high-throughput, automated sequence sorting algorithm;
2. The systematic collection of scientific literature supporting the identification of allergens;
3. The coordinated review by an external peer review panel (PRP) of internationally recognized allergy experts from the public sector;
4. An annual public release of the database by repeating steps 1–3 to identify new allergens; and
5. Independent management and documentation practices by the non-profit scientific organization HESI, that ensures the integrity of the database and transparency of the process used to populate the database (e.g., “Documentation” link in the database page, which comprises important information about the content of updates, new features or list of upgrades accompanying a new database release as well as a “COMPARE transparency document” listing all entries reviewed by PRP, their comments and decisions).

Detailed descriptions of each of these procedures (scientific rationale, methodological approach, and assurance of balance/transparency) follow below.

### Development of a High-Throughput, Automated Sequence Sorting Algorithm

#### The Pilot Process Development and Validation

Presently, three primary databases contain all the existing sequence data so far generated (GenBank, EMBL database and DNA Data Bank of Japan). As these databanks are interconnected through the International Nucleotide Sequence Database Collaboration (http://www.insdc.org/), data submitted to any one of these databases are shared by, and hence can be retrieved from, all three.

The starting point for the COMPARE screening process is the National Center for Biotechnology Information (NCBI) Protein sequence database (https://www.ncbi.nlm.nih.gov/protein), which includes translations from annotated coding regions in GenBank, RefSeq and TPA, as well as records from SwissProt, PIR, PRF, and PDB.

COMPARE's initial pilot search was designed in 2016 to identify NCBI Protein sequence entries that contain the term “allerg^*^” (which encompasses allergen, allergy, allergenic, and allergic) and include proteins that are derived from animals, plants, fungi, and protists. This pilot search of the NCBI Protein sequence database was completed through the main query window (https://www.ncbi.nlm.nih.gov/protein) using the following Boolean search:

“allerg^*^ AND [time period: from Jan 1, 2014 to Dec 31, 2014 or Jan 1, 2015 to Dec 31, 2015] AND [species: animals, plants, fungi and protists].”

By design, the initial search term “allerg^*^” is very broad, can be found anywhere in each GenPept record, and does not necessarily relate to the identification of a protein allergen. Extended search criteria were thus applied in a stepwise fashion to further refine the broad “allerg^*^” search results. Keywords for filtering were selected by informatics, allergy, toxicology, and clinical experts (participating members of the public-private COMPARE Steering Team) through an iterative observation-based process, taking into account contextual categorical features such as the sequence's source organism, whether the sequence was submitted through an automated annotation pipeline (i.e., genome sequencing projects), whether “allerg^*^” appeared in a protein definition description, and feature lines such as ‘/note = “allergenic/antifungal thaumatin-like proteins.”

Manual examination of random subsets of the search results at various points of the filter helped identify additional keyword-based rules capable of distinguishing between potential allergen candidates (those related to allergenicity and submitted to the expert panel for review) and irrelevant entries (those unrelated to allergenicity and filtered out to reduce the “noise” in the review). These rules were evaluated and compared for their efficiency to eliminate non-allergen entries and the potential for overly aggressive exclusion, which might lead to undesirable omission of potential allergen candidates. Of the two to five rules evaluated in each step, the rule with the highest efficiency and lowest potential for overly discriminate exclusion was selected. When more than one rule had comparably high efficiency, preference was given to the one with a lower chance of omitting potential allergen entries.

#### Testing and Validation of the Filtering Algorithm

The COMPARE process was validated by testing it against the AllergenOnline v.16 database. The results indicated that many of the sequences containing “allerg^*^” in their annotation were classified as *candidates* by the COMPARE algorithm, as expected. Yet this testing also found that some sequences in AllergenOnline v.16 (GenPept format) do not contain the term “allerg^*^” in their annotation, and as such were not being captured. Manual examination of those sequences revealed that they are typically associated with specific allergen designations such as profilin or tropomyosin. This validation step allowed the identification of additional keywords (Supplementary Table 1) to be used in parallel to the “allerg^*^” search of the NCBI Protein database. This supplemental search and filtering was performed for the 2017 and 2018 COMPARE database builds. After conducting the supplemental search in parallel of the main search for 2 years, it appeared that the results did not produce significantly new information compared to the outcome of the main algorithm search. This parallel approach was therefore discontinued in subsequent years.

The final COMPARE keyword-based filter consisted of 13 steps and 28 elements (i.e., decision steps/points) (Supplementary Figure 1).

### Continuous Improvement Strategies

#### Additional Sources of Allergen “Candidates”

A robust search for potential new allergen candidates needs to cast a broader net than a year's worth of NCBI Protein database entries for several reasons. First, an allergen may have been identified but not submitted to the NCBI Protein database. This is likely to occur when the allergen discovery is extremely recent. Second, there may be older entries in the NCBI Protein database for which new evidence of allergenicity may have become available. A solution to both situations is a thorough search of the scientific literature each year to capture new evidence. Such a process was implemented in the development of COMPARE 2018 and future iterations of the database, the parallel direct literature search strategy used is described in (Appendix-1 in Supplementary Materials). Lastly, there are other important protein sequence databases available to the scientific community. For example, UniProtKB is a protein database partially hand-curated by experts (26, 27), it was included in the bioinformatics screening for identification of candidate allergens as of COMPARE 2019. There are other allergen-specific databases with various degrees of overlap, as reviewed by Radauer (16) and Radauer and Breiteneder (15), from which new allergen candidates not included in COMPARE could be evaluated by the COMPARE expert panel. Such databases include the World Health Organization (WHO)/International Union of Immunological Societies (IUIS) Allergen Nomenclature Database (http://www.allergen.org/) (6), responsible for maintaining and developing a unique, unambiguous, and systematic nomenclature for allergenic proteins, and AllergenOnline (http://www.allergenonline.org) (8), as both are updated regularly (on an ongoing basis and yearly, respectively). As part of 2017' and 2018' workstreams, and a commitment to continuous improvement, the COMPARE process incorporated progressively new sources of allergen candidates (Table 1).

**Table 1:** COMPARE database year-by-year evolution of the process development and resulting end-products.

**Process**		**End product**		
**Calendar year**	**Development stages and sources** **of candidate allergens**	**Database version**	**Release date**	**Number of entries**
2016	Project launch: pilot process development and validation.	COMPARE 2017	February 3, 2017	1,970^a^
	Algorithm screening: “Allerg*”-based and specific keyword-based, applied to NCBI's Protein database.			
2017	Improved algorithm and extended list of keywords (Supplementary Figure 1; Supplementary Table 1) applied to NCBI's Protein database.	COMPARE 2018	February 16, 2018	2,038
	New source for candidate allergen identification incorporated in the COMPARE process (in addition to the bioinformatics screenings), via a separate targeted literature search.			
	Sequences from IUIS and AllergenOnline not present in COMPARE 2017 were added to the list of “Candidate Sequences,” along with their respective supporting references, for review by COMPARE's panel of experts.			
2018	Updated bioinformatics screening method. Bioinformatics screening applied to UniProt, in addition to NCBI's Protein database.	COMPARE 2019	January 18, 2019	2,081
	Separate targeted literature search. Sequences (with respective literature) from IUIS and AllergenOnline not present in COMPARE 2018 were included in the list of “Candidate Sequences.”			
2019	“Historical screenings”—a plan to undertake a historic screening by applying the COMPARE process to sequence records dated from 2016 and previous years was completed. This project was undertaken to harmonize the database content to up-to-date COMPARE processes, given that the COMPARE process was developed in 2016 and applied only to a “1-year” time window every year since then. “Database audit”—this process was conducted by an external Bioinformatics services provider to analyze the current content of the COMPARE database as a quality control measure.	COMPARE 2020	January 29, 2020	2,248
2020	A “Parent accession” field was added in the details view page of entries. This field applies particularly to smaller fragments, derived from mass-spectrometry studies, and is intended to connect the fragment to the full protein from which it is derived (when indicated in the literature associated). A “parent accession” number will be indicated in that field when applicable and when available (not all entries will have one). The parent accession is NOT an entry in the database and merely provided as additional metadata for users' reference.	COMPARE 2021	January 29, 2021	2,348

*COMPARE, COMprehensive Protein Allergen REsource; IUIS, World Health Organization/International Union of Immunological Societies Allergen Nomenclature Database; NCBI, National Center for Biotechnology Information*.

a *The first version of COMPARE was built from the pre-existing AllergenOnline v.16 database (1,956 entries; http://www.allergenonline.org), to which 14 new entries approved by the COMPARE peer review panel were added*.

#### Incorporation of Sequence Comparison Capabilities Accessible to COMPARE Users

A key to understanding cross-reactivity from a sequence-based perspective is to identify a minimum degree of similarity between a *query protein* and a *known allergen* that can prompt further investigation. Global guidelines on a minimum similarity (e.g., >35% sequence identity over a sliding window of at least 80 amino acids) have evolved in several ways (28–35) and the validity of the various criteria has been discussed in various reviews (36–41). However, while the scientific merits of these criteria are still evaluated and discussed, all approaches rely on a common element: a database containing protein sequences of all currently recognized allergens, to enable *in silico* processes of assessing a protein's potential allergenicity.

In January 2019, COMPARE 2019 was released with a more dynamic interface to facilitate the ease of access to relevant information to all users (clickable links for easier access to literature sources) and for increased functionality. This new interface allowed the incorporation of a built-in FASTA-based sequence comparison tools, “COMPASS” (COMPare Analysis of Sequences with Software), where users can compare their selected (query) protein sequence to the allergens in the database. The software of choice has traditionally been the FASTA algorithm (42) and is recommended for assessing similarity between protein sequences. Therefore, COMPASS operates with the open source FASTA software package (FASTA v36 at the time of this publication; https://fasta.bioch.virginia.edu/fasta_www2/fasta_list2.shtml). The incorporation of FASTA search algorithm as the core of the COMPASS search tool on the COMPARE database supports real-time sequence comparisons using the following three approaches without downloading the database and installing software on local computers/servers: (1) full-length sequence comparison; (2) 80-aa (amino acids) sliding window comparison; (3) 8-aa exact match [based on FAO/WHO (30) and CODEX Alimentarius Commission (28, 43) guidelines on the evaluation of proteins derived from modern biotechnology for allergenicity]. As of July 2020, the COMPASS search tool offers a visualization component, allowing users to view results in a graphical display. Additionally, other features such as a reference list and synopses of relevant literature and guidelines, as well as a sort-and-search function of the database listing are available for educational purposes in the COMPASS homepage and “About” tab.

### Literature Procurement

One of the key requirements of the COMPARE process is that a “candidate allergen sequence” retained for review by the expert panel has at least one supporting scientific publication related to the specific candidate sequence. This rule is strictly applied in COMPARE, including in cases where a sequence is listed in IUIS but a publication is not yet available (e.g., Der p 37, Der f 26 and Per a 13). These entries are accepted by IUIS based on information provided in direct communication between the submitters of the allergen and the IUIS committee. In relation to other databases, for example, this may also explain differences between COMPARE and AllergenOnline, discussed in section 4.1. Examples of entries in COMPARE not found in IUIS or AllergenOnline include QCI56569.1, Q9YGJ8.2, and CAA58223.1. The identification and procurement of primary scientific literature that relates to the sequences identified as new “allergen candidates” is the second step in the COMPARE process, after the bioinformatics screenings and parallel direct literature search (Appendix-1 in Supplementary Materials). JIFSAN collaborates in the COMPARE process by facilitating (among other program components) the procurement of the literature associated with the candidate allergens and administrating a custom-built web-based portal where the candidate-specific information from all sources is integrated for peer review by an independent panel of international experts.

### Peer Review Process

Candidate allergens and associated primary literature data are provided to the COMPARE PRP. The scientific literature review process and voting is the exclusive purview of the PRP and is conducted independently of other COMPARE stakeholder involvement. The PRP determines whether the candidate protein sequence has enough supporting evidence of allergenicity to be included in COMPARE, based on a documented set of criteria (described below) to determine the quality and extent of the data presented in the literature upon which a decision to include/exclude a candidate sequence can be based.

The COMPARE PRP is an international group of five academic and clinical allergy experts (see list of authors' contributions for names of experts in the panel) renowned for their research and expertise in areas such as the nature of protein allergenicity and molecular characterization of allergens, allergenic cross-reactivity, immune-based mechanisms of allergy, immunotherapy, allergen diagnostics, and component-resolved diagnosis. The panel participants operate as volunteers with support from HESI's scientific program management staff and from JIFSAN's staff for information management of the sequences, associated literature data, and implementation of a custom system to collect decisions from PRP experts (PRP review tool). PRP members receive a nominal honorarium via HESI.

#### Criteria for Inclusion and Exclusion of Allergens in COMPARE

The PRP evaluation criteria are consistent with those widely adopted for inclusion of sequences in other allergen databases (8, 44, 45). The minimum criterion adopted for inclusion of a candidate is peer-reviewed evidence of IgE binding, either in the published literature or other peer-reviewed documentation. This criterion is highly conservative, with no requirement for demonstration of IgE functionality (e.g., cross-linking for degranulation of IgE-bearing cells).

The evaluation of evidence for IgE binding includes consideration of the experimental approach, the quality of sera, and characterization of the IgE binding itself. Ideally, IgE binding is demonstrated to a purified full-length recombinant version of the allergen. In case evidence of IgE binding is based on purified natural protein, the peer-review panel gives special attention to the evidence provided with respect to purity on a case-by-case basis. Similarly, if evidence of IgE binding is based on proteomic approaches combining IgE 1D- or 2D-blots and mass spectrometry, single bands or spots should be discrete enough and each contain sequence evidence from one protein only. Peptides (10 amino acids in length or above) explicitly identified by mass spectrometry and which sequences are included in the supporting literature are accepted as individual entries. As a result, a single protein may be represented in the database by several peptide fragments, listed as individual entries. Table 2 summarizes the inclusion and exclusion criteria, as well as considerations for the scientific review decision process, defined by the COMPARE PRP. Table 3 is a concise overview of the criteria used to declare an allergen in the COMPARE database.

**Table 2:** COMPARE peer review panel: scientific review decision process and inclusion/exclusion criteria defined by the experts in the independent panel.

**Inclusion criteria**	**Exclusion criteria**
• Minimum criterion: peer-reviewed evidence of IgE binding; either in the published literature or other peer-reviewed documentation. • Highly conservative; no requirement for demonstration of IgE functionality (e.g., cross-linking for degranulation of IgE-bearing cells). • Data quality evaluation ◦ *Experimental approach:* ▪ Use of well-established or validated assay(s). If not, a well-described design is necessary. ▪ Presence of appropriate negative controls. ◦ *Serum quality:* ▪ **Best:** sera from patients with proven sensitization and/or reported allergy to the source of the candidate protein sequence. ▪ **Viable option:** sera from patients proven allergic to a source likely to be cross-reactive with the source of the candidate entry. ▪ If sera contain high total IgE levels (e.g., from patients with atopic dermatitis or with parasite infections), appropriate control sera with high total IgE need to be included. ◦ *Nature of the IgE binding: cross-reactivity to CCDs and/or galactose-αGAL:* ▪ If there is indication that IgE binding may exclusively be directed toward glycan moieties on the tested protein (natural purified glycoprotein or recombinant eukaryotic glycoproteins), other proof of IgE binding, specific to the protein backbone, needs to be provided (e.g., inhibition studies to exclude the possibility that IgE is directed only toward glycan moieties on the protein). ◦ *Use of natural purified proteins to demonstrate IgE binding:* 1. The purity and the impact of potential contamination with traces of known allergens needs to be addressed in the supporting literature for consideration.	• Lack of appropriate negative control sera. • Insufficient protein purity. • Probable IgE binding exclusively to carbohydrate determinants (not to protein backbone)^a^; i.e., absence of evidence that pre-absorption or inhibition with a homologous non-glycosylated peptide or polypeptide decreases IgE binding to the protein with appropriate controls. ◦ Full inhibition of IgE binding by relevant carbohydrate structures (CCD/αGAL) • Homologs to known allergen sequences without supporting published evidence: homology alone without published evidence of allergenicity associated with the specific protein sequence does not warrant inclusion in COMPARE.

*αGAL, alpha-1,3-galactose; CCD, carbohydrate determinant; COMPARE, COMprehensive Protein Allergen REsource*.

a *Exclusive IgE binding to CCD is considered a “false positive” as it is not related to the protein sequence (46)*.

**Table 3:** COMPARE database criteria for an allergen sequence.

**Allergens are included in COMPARE when they meet all three of the following criteria:**
1. Peer reviewed publication
2. Evidence of sequence data—full length, partial sequence (if coverage is convincing)
3. IgE binding activity (from human sera; no veterinary allergens included up to now; carbohydrate epitopes excluded up to now)

Candidate sequences rejected by the panel for lack of evidence can be revisited in subsequent years if new evidence becomes available.

Information regarding all candidate allergens submitted to the PRP each year, whether ultimately “accepted” or “rejected,” are retained in internal records for tracking purposes. With the release of COMPARE 2019, the reviewers' decisions and comments about all “accepted” or “rejected” candidates were made publicly available as part of the COMPARE transparency documentation (see the “Database” page and “Documentation” tab at http://www.comparedatabase.org).

**Figure 1:**
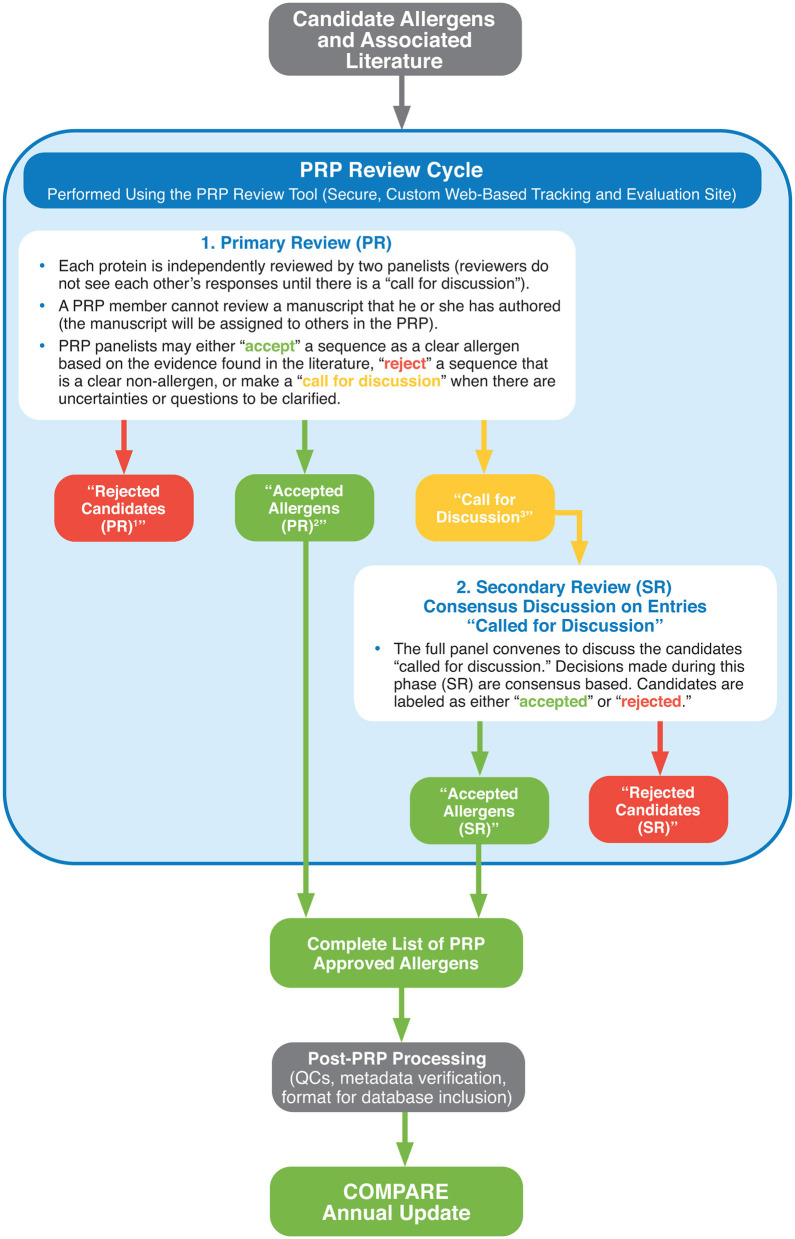
Summary of the COMPARE Peer Review Cycle and Workflow for each annual update.

In summary, Figure 1 provides a description of the COMPARE peer review cycle and workflow applied at each annual update.

Note that since COMPARE started building upon the existing AllergenOnline v.16 database, allergens with an entry date of 2016 or prior years were not subjected to the COMPARE peer review process.

### Maintaining Data Currency and Accuracy

A sequence database that is used to identify potential hazards related to food safety is of maximum utility if it contains the most recent allergens. Therefore, COMPARE maintains its utility via an annual screening of protein sequence databases (NCBI Protein, UniProtKB) and other sources (as described in sections 2.4 and 2.5). It is expected that the COMPARE process will continue to evolve each year, to adapt to the changes in sequencing technology and informatics/annotation trends observed in a previous update cycle. An annual update process facilitates confidence in communicating the presence of new allergens in a timely fashion, while still allowing for a thorough, high-quality review process. The established cyclic annual workflow supports a January (of the next new year) release of the updated database with sequence culling from public databases conducted early enough in the year (typically from May to May).

COMPARE's open processes provide continued opportunities to ensure that the most contemporary evidence is evaluated and incorporated. The COMPARE website includes a dedicated “Contact Us” tab (https://comparedatabase.org/contact-us/) and any user can submit comments through the website portal or the contact email provided.

## Implementation and Results

To date, the COMPARE process as described above, with some limited modifications, has been implemented to produce the first release of the database (COMPARE 2017) and four subsequent annual updates (COMPARE 2018–2021 data releases; Table 1). The web interface presents the database main fields in a searchable table view with the following fields for each allergen sequence entry: Species (of the source organism); Common Name, Description (of the allergen sequence), IUIS Name, Accession, Length, Year Adopted (the year of inclusion in the database—*note that for sequences marked “2016” or years prior, this corresponds to the year listed for entry of the sequence in AllergenOnline, the foundational data from which COMPARE developed, as marked in AllergenOnline v.16*), and a “VIEW” clickable box leading to a window with the individual data for each sequence (the actual sequence, and supporting scientific publications with evidence of allergenicity).

**Figure 2:**
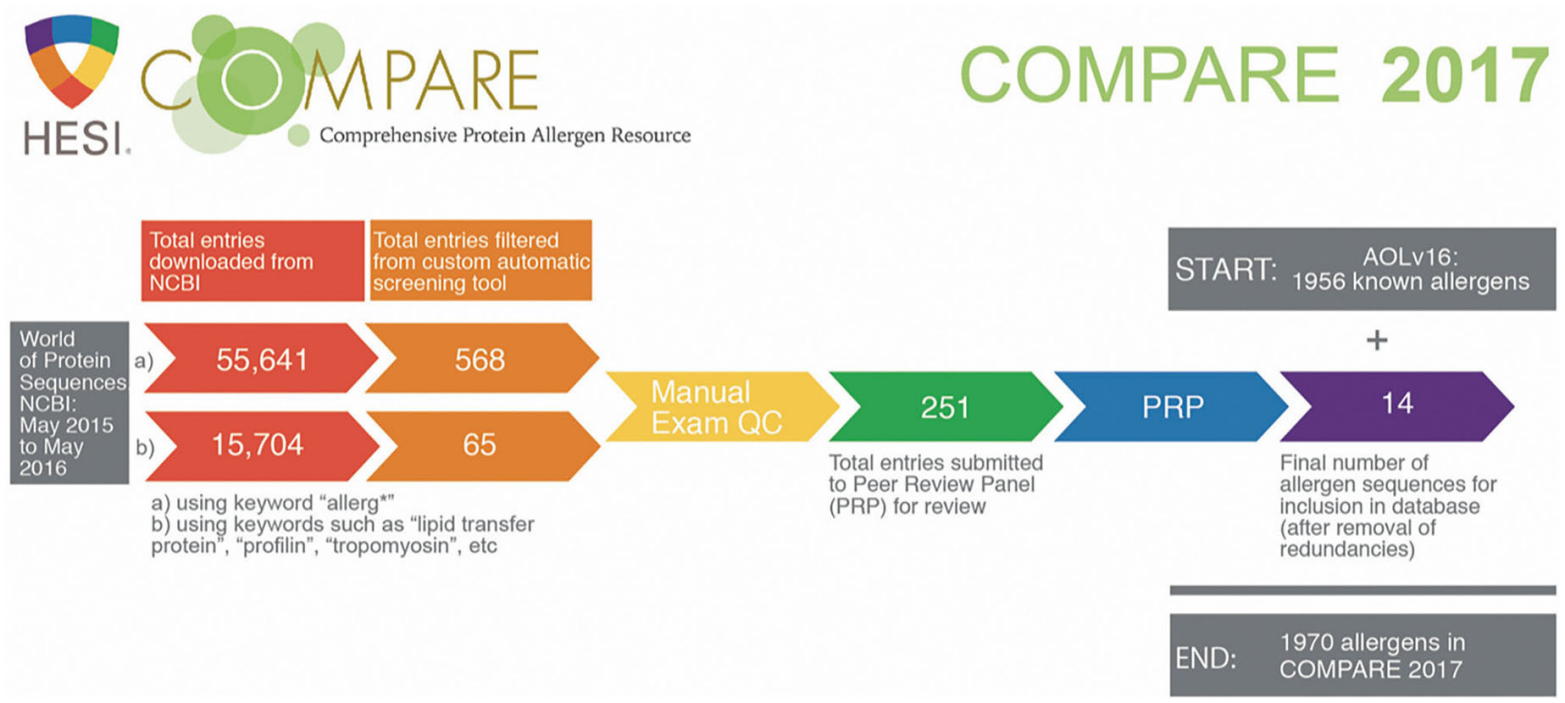
COMPARE 2017 screening process quantitative outcomes (supporting information provided in Appendix-2 of the Supplementary Materials).

Figure 2 provides an overview of the 2017 COMPARE database build process and quantitative outcomes of the screening process. Candidate entries for the 2017 COMPARE database were obtained from the NCBI Protein database as accessed through the main query window (https://www.ncbi.nlm.nih.gov/protein) on May 14, 2016 using the following Boolean search:

“allerg^*^ AND [time period: from May 30, 2015 to May 14, 2016] AND [species: animals, plants, fungi and protists].”

The original methodology was improved by broadening the sources of sequences to include IUIS and AllergenOnline as new sources, in addition to the NCBI Protein database. Therefore, sequences under the IUIS listing, going back to June 2015, were evaluated in the build of COMPARE 2018, and any sequences present in AllergenOnline v.17 that were not already included in COMPARE 2017 were evaluated for potential inclusion. Additionally, a targeted peer-reviewed literature search was implemented to identify new allergen sequences (Appendix-1 in Supplementary Materials). Supplementary Table 2 describes the number of candidate allergens identified through the various sources and the final number of allergens accepted in COMPARE 2018, from each category. The 2018 COMPARE database included 68 allergen sequences added to the 1,970 allergens constituting COMPARE 2017 (2,038 total).

The COMPARE 2019 evaluation process results are presented in Supplementary Table 3; there are 2,081 sequences in COMPARE 2019.

The COMPARE 2020 evaluation process results are presented in Supplementary Table 4; there are 2,248 sequences in COMPARE 2020.

The COMPARE 2021 evaluation process results are presented in Supplementary Table 5; there are 2,348 sequences in COMPARE 2021.

Along with the continuous database update and process improvement, sequence comparison tools associated with the database were implemented to provide bioinformatics analysis of proteins for potential allergenicity. Currently, there are three sequence comparison approaches accessible to the public: (1) FASTA search of the whole protein sequence as a query against the up-to-date COMPARE database. The search parameters such as E-value can be set by the user. The output alignments can be downloaded for further evaluation; (2) FASTA searches (default search parameters) of each sequentially overlapping 80aa fragment (sliding window) from a query protein against the up-to-date COMPARE database using the default search parameters of FASTA. Any alignment >35% identity over ≥ 80 aa will be displayed as a hit that needs further evaluation based on the guideline of FAO/WHO (30) and CODEX Alimentarius Commission (28, 43); (3) A FASTA search (default search parameters) of the whole protein sequence as a query against the up-to-date COMPARE database for the exact match of eight contiguous amino acids with the allergens in the database, although this approach adds little value to the risk assessment of proteins for potential allergenicity due to high false positive rates (47–49).

## Discussion and Strategies for Harmonization

Reliable resources to assess the allergenic potential of novel proteins expressed in foods are essential to confidently make safety determinations and provide robust resources with public accessibility. The internationally recognized standard for safety assessment of foods derived from biotechnology is the Codex Alimentarius Commission (43) and recommends that novel proteins expressed in foods be analyzed for any amino acid sequence similarities to known allergens. Using a comparative sequence-level approach for the initial scan of potential allergens allows for a reproducible and fully documented process to establish a protein allergen database. Clinical relevance of allergen sequences by a panel of scientific experts in allergology provides validation that entries added in COMPARE are known allergens, allowing the database to be a reliable resource. There are many constituencies that may be interested in this type of database: product developers providing safety information on novel proteins; regulatory agencies responsible for food and feed safety assessments; medical personnel in the allergy field; the public, who may be interested in identifying sources of allergens. Overall, the transparent and peer-reviewed method to generate the COMPARE database continues to meet the requirements of regulatory agencies to communicate the output results of protein allergenicity screens to the public.

### Learnings From 5 Years of COMPARE

The results portrayed in Figure 2 (quantitative outcomes from the screening and review processes for COMPARE 2017) illustrate how important it is to implement an automated sorting algorithm to sort through the vast amount of “noise” retrieved from protein sequence database screenings, when searching for any protein related to “allerg^*^” (55,641 hit sequences) or when using keywords very specific to allergens such as profilins, tropomyosins, and so on (15,704 hit sequences). The analysis of the results of the second-year process suggests that the noise increases year by year: the combined retrieved numbers for “allerg^*^” and specific allergen keywords in the initial screening were close to 118,000 hits for COMPARE 2018, almost double that for COMPARE 2017 (data not shown).

Furthermore, the analysis of the number of candidate allergens identified through the various sources compared to the final number of allergens accepted from COMPARE 2018 to 2021 (Supplementary Tables 2–5), for each category, sheds light on questions about the differences between the total number of allergens in COMPARE and AllergenOnline. In fact, some AllergenOnline candidate entries do not meet the COMPARE criteria: in most years the PRP rejected around 50% or more of the candidate sequences from AllergenOnline, on the basis that the articles associated with those entries in AllergenOnline did not contain the specific sequence being evaluated; the IgE binding data provided in the article was not related to the exact candidate sequence; or the candidate sequence and the data in the associated article referred to different species. In other words, the requirement of an exact match between the candidate sequence being evaluated and the sequence(s) tested in the associated literature was not met in these specific instances (specific examples can be found in the COMPARE Database webpage, by clicking on the “Documentation” hyperlink, and then opening the “Transparency file”).

Additionally, the standardized key-word search of literature databases has proven to be a valuable complementary source of valid candidates and resulted in some instances in additional allergens being picked up by COMPARE that were not listed in AllergenOnline of the same year's update. Examples of this include accessions ADV71357.1, NP_001036878.1, NP_001138311.1, XP_392204.2, which are not found in AllergenOnline at the time of these writings.

### Strategies for Harmonization

Given the scientific and public health value of a curated allergen database, opportunities to continually enhance its utility are paramount. As such, several improvements have been identified and have been completed or are under active development. These activities (further described in the paragraphs below) include: (1) retrospective historic screenings of the years prior to 2016 to identify possible extra allergens that may not have been included in AllergenOnline v.16, and (2) curation of the COMPARE database to include all available WHO/IUIS recognized allergen designations.

#### Applying Current COMPARE Approaches to Historical Datasets

As described in this article, COMPARE builds on the foundational sequence list developed over a 10-year period by the University of Nebraska FARRP, which publishes AllergenOnline (8). The list of sequences from AllergenOnline v16 were imported “as is” and were not evaluated by COMPARE PRP. Recently, a screening of historic datasets with records dated from 2016 and years prior was undertaken by applying the COMPARE process to harmonize the database content to up-to-date COMPARE processes (since from the beginning, starting in 2016, the process always consisted in screenings of the previous “1 year” time-window). In this process, the decision was taken to not remove sequences uploaded into COMPARE 2017 from the foundational sequence list from AllergenOnline v.16, but only to complement the database with additional sequences identified using the COMPARE algorithm applied retrospectively and reviewed by PRP. Therefore, the data from AllergenOnline v.16 has remained intact over the years, as imported for the built of COMPARE 2017. This choice was made to remain conservative and to avoid confusion amongst users. Overall, based on the high-throughput COMPARE screening process, and after PRP review, the historic screenings contributed to 57 new sequences added in COMPARE 2020, a substantial part of them not included in the current version of AllergenOnline. The supporting articles associated with these sequences are dated from 2016 or before, and it is unclear why they were not part of AllergenOnline v16. Sequences from the historic screenings are labeled as “2020H” or “2020H_MS” in the field “year adopted” of the database, as well as in column B of the “COMPARE-2020 Transparency file” (available under the “Documentation” tab of the COMPARE database website), to distinguish them from entries resulting from the regular annual screening process, labeled “2020”.

#### Curating Allergen Designations in COMPARE to Nomenclature Standards

Standardized allergen naming conventions, as set by the WHO/IUIS Allergen Nomenclature Sub-Committee, help reduce the use of non-standard allergen names and descriptions and are a key aspect of understanding related proteins across species (6). Unfortunately, not all sequence records of allergens comply with IUIS standards: allergens identified in older research have been named according to various non-standard conventions; many allergen sequences identified in more recent years often include irrelevant automated annotations from sequence records. For these reasons, it is important to undertake a curation of allergen designations to generate meaningful description lines to the eyes of allergy experts and allergen database users, based on official IUIS allergen designations when available, as opposed to automatically adopting description lines from source protein records (generally from the NCBI Protein database or UniProtKB). Such curation has been started in COMPARE 2019 (for all new entries added in 2019) based on a defined stepwise priority approach described in the “COMPARE 2019 Documentation File” (available at http://db.comparedatabase.org/docs/COMPARE-2019-Documentation-2019-01-17.pdf?v=20190117), and has continued in future iterations of COMPARE. The COMPARE management team expects the adoption of allergen names to current standards, as set by the WHO/IUIS Allergen Nomenclature Sub-Committee (http://www.allergen.org), to be an ongoing quality improvement process that will occur in parallel with the curation of the “Description” fields, by the experts in the PRP. The goal will be to alleviate confusion over multiple names and make clearer the link between the publications that support a protein's inclusion in COMPARE as an allergen and sequences themselves.

## Conclusions

The COMPARE database provides a rigorous, transparent, and annually updated resource for screening potential allergens. The data-driven and clinical research–based screening processes allow for comprehensive and clearly documented sequence identification and evaluation by an independent panel of experts. The process has flexibility to account for changes in technology, variability in terminology, and annotation trends by incorporating the observations and learnings of a given cycle into the following build cycle, reflecting ongoing continuous improvement. With oversight and coordination by HESI, additional enhancements to COMPARE are anticipated to provide added utility to meet contemporary safety assessment and research needs into the future. In addition, the bioinformatics analytical capability through COMPASS provides tools to the public for the risk assessment of potential protein allergenicity.

## Data Availability Statement

The COMPARE database is publicly available at: http://www.comparedatabase.org. Bioinformatics analytical tools (COMPASS) associated with the COMPARE allergen database are publicly available directly from the database page, or via: https://comparefasta.foodrisk.org/. The original contributions presented in the study are included in the article and Supplementary Material, further inquiries can be directed to the corresponding author.

## Author Contributions

RR: writing/critically reviewing of manuscript, COMPARE steering team member, and peer-review panel chair. DS: programmatic support for: database design, design & development of online tool for peer-review panel, development of the COMPASS tool, and critically reviewing of manuscript. MB: critically reviewing of manuscript, peer-review panel member (until 2019 update, replaced by GG of the University of Salzburg). LB, SM, AS, PS, and EI: writing/critically reviewing of manuscript, COMPARE steering team member (scientific & technical advisory). AC: programmatic support for: database design, design & development of online tool for peer-review panel, development of the COMPASS tool, and critically reviewing of manuscript. GG: critically reviewing of manuscript and peer-review panel member. PG: programmatic support for: database design, design & development of online tool for peer-review panel, and critically reviewing of manuscript. KH-S: critically reviewing of manuscript and peer-review panel member. LK: critically reviewing of manuscript and COMPARE program manager. JK and GL: critically reviewing of manuscript and COMPARE steering team member (scientific & technical advisory). KM: programmatic support for: database design, design and development of online tool for peer-review panel, development of the COMPASS tool, and critically reviewing of manuscript. SM-R: critically reviewing of manuscript and COMPARE program manager. CN: critically reviewing of manuscript and design/construction online tool for peer-review panel. LPM: writing/critically reviewing of manuscript and COMPARE program manager. SP: project design and stakeholders' involvement and critically reviewing of manuscript. LKP: critically reviewing of manuscript, COMPARE steering team member, and peer-review panel member. ST: critically reviewing of manuscript and peer-review panel member. MB: writing/critically reviewing of manuscript and COMPARE steering team member (2016–2017). All authors contributed to the article and approved the submitted version.

## Conflict of Interest

LB was employee of Limagrain Field Seeds. EI was employee of BASF Corporation. GL was employee of DuPont Co., Nutrition and Biosciences. SM was employee of Syngenta Crop protection. AS was employee of Bayer Crop Science. PS was employee of Corteva Agriscience LLC. CB was employed by Bayer Crop Science. GS was employed by company IFF Inc. The remaining authors declare that the research was conducted in the absence of any commercial or financial relationships that could be construed as a potential conflict of interest.

## Publisher's Note

All claims expressed in this article are solely those of the authors and do not necessarily represent those of their affiliated organizations, or those of the publisher, the editors and the reviewers. Any product that may be evaluated in this article, or claim that may be made by its manufacturer, is not guaranteed or endorsed by the publisher.
