# Supplementary material for: The COMPARE Database: A Public Resource for Allergen Identification, Adapted for Continuous Improvement
Source: Front Allergy. 2021 Aug 6;2:700533. doi: 10.3389/falgy.2021.700533 (PMC8974746; doi:10.3389/falgy.2021.700533)
Supplement: Supplementary file 1 [file Data_Sheet_1.pdf]

Supplementary Materials - COMPARE Database Manuscript, Ree et al.

“The COMPARE database: a public resource for allergen identification, adapted for continuous improvement”.

Supplementary Figure S1. Custom COMPARE automatic keyword-based filter designed to identify candidate allergens from the NCBI Protein sequence database. The filter consists of 13 consecutive steps and 28 elements (i.e., decision steps/points).

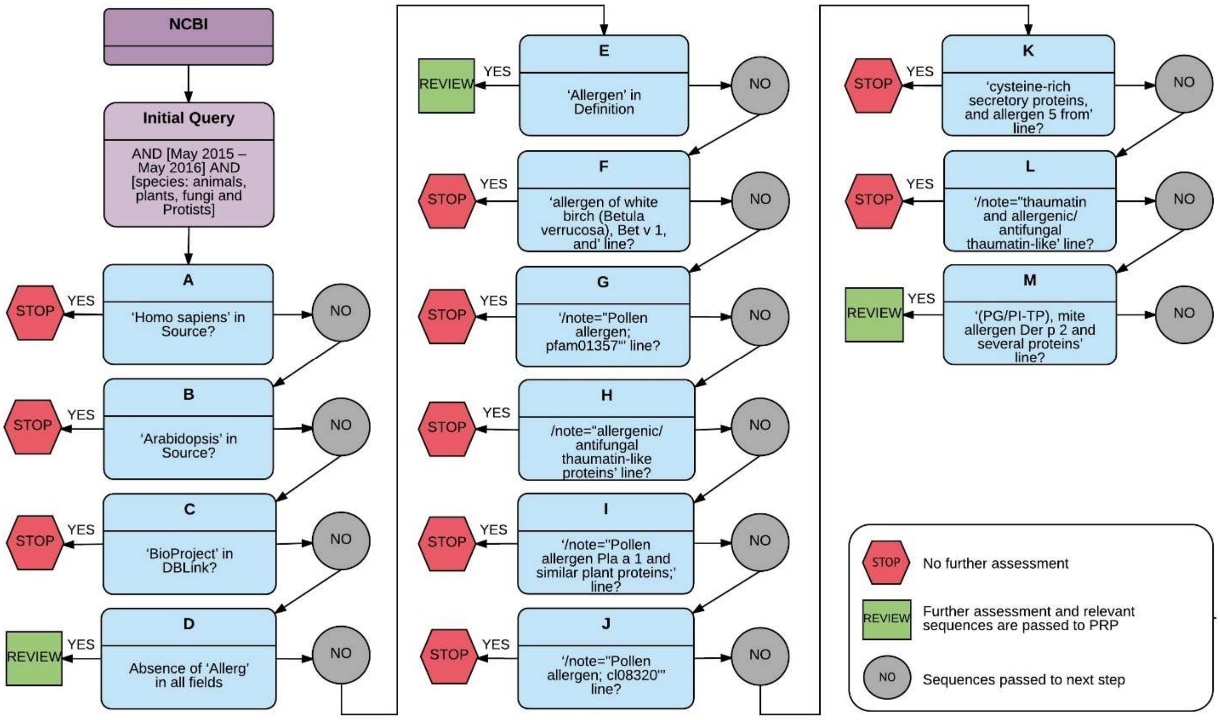

A key filter is based on the source species. For instance, at elements A and B, source species "*Homo sapiens*" and "*Arabidopsis thaliana*" were excluded because they were not considered a relevant source of allergens (this decision is currently being revisited for future versions of COMPARE, as described in section 2.3.1). Other rules identify features indicative of high-throughput sequencing studies where evidence of allergic reaction obtained through human allergic serum IgE testing would not be present. For example, at element C, the presence of keyword “BioProject” in the DBLink field is a strong indication that the entries resulted from a high-throughput study (which could be one of several types, including genome sequencing and assembly, metagenome, transcriptome sequencing and expression, targeted locus sequencing, etc.).

<u>Process update:</u> Starting in 2019 (i.e., building of COMPARE 2020), the exclusion filter on the basis of the organisms “*Homo sapiens*” and “*Arabidopsis*” was removed at the bioinformatics screening stage, per

decision of the PRP, out of concern for missing eventual allergens newly discovered from these two species (*e.g.,* there is growing evidence of the existence of IgE against human proteins or self-reactive IgE in patients with autoimmune disorders).

Supplementary Table S1. Specific keywords for supplemental searches

Defined keywords used in searches to identify protein sequences without "allerg*"	
11S globulin	glycinin
2S albumin	lipid transfer protein
7S globulin	lipophilin
8S globulin	oleosin
agglutinin	patatin
alpha-amylase	profilin
beta conglycinin	proteinase inhibitor
calcium binding protein	serine protease
casein	serpin
chitinase	transaldolase
collagen	tropomyosin
convicilin	trypsin inhibitor
enolase	vicilin
glucananse	vitellogenin

Supplementary Table S2. Quantitative outcomes of the COMPARE 2018 screening and evaluation process

Source	Reviewed allergens	Reviewed articles	Approved allergens
Bioinformatics screenings (NCBI Protein database)	93	186	27
Literature search	-	37	29
AllergenOnline	22	35	12
**Total**	115	258	**68**

The table describes the number of candidate allergens identified through the various sources and the final number of allergens accepted in COMPARE 2018, for each category, after peer review panel review.

Abbreviations: AOL, AllergenOnline; COMPARE, COMprehensive Protein Allergen REsource; IUIS, World Health Organization/International Union of Immunological Societies Allergen Nomenclature Database; NCBI, National Center for Biotechnology Information.

Supplementary Table S3. Quantitative outcomes of the COMPARE 2019 screening and evaluation process

Source	Reviewed "candidate allergens"	Reviewed articles	Approved allergens
AllergenOnline	29	75	13
AllergenOnline, NCBI Protein	3	1	0
AllergenOnline, NCBI Protein, UniProt	2	1	2
NCBI Protein	7	7	1
NCBI Protein, Literature search	2	2	1
NCBI Protein, UniProt	34	6	9
IUIS	17	1	1
Literature search	76	27	21
Other	1	1	1
UniProt	10	12	1
**Total**	181	133	**50**

The table describes the number of candidate allergens identified through the various sources and the final number of allergens accepted in COMPARE 2019, for each category, after peer review panel review.

Abbreviations: COMPARE, COMprehensive Protein Allergen REsource; IUIS, World Health Organization/International Union of Immunological Societies Allergen Nomenclature Database.

Supplementary Table S4. Quantitative outcomes of the COMPARE 2020 screening* and evaluation process

Source	Reviewed "candidate allergens"	Reviewed articles	Approved allergens
AllergenOnline	92	16	9
AllergenOnline, IUIS	3	2	0
IUIS	13	9	10
NCBI Protein	9	2	5
NCBI Protein, UniProt	1	1	0
Literature search	71	33	51
Other	3	2	3
UniProt	9	6	5
**Total**	201	71	**83**

The table describes the number of candidate allergens identified through the various sources and the final number of allergens accepted in COMPARE 2020, for each category, after peer review panel review.

Abbreviations: COMPARE, COMprehensive Protein Allergen REsource; IUIS, World Health Organization/International Union of Immunological Societies Allergen Nomenclature Database.

*Does not include the one-time "Historical screening" entries included in COMPARE 2020 (available upon request and in the COMPARE 2020 Documentation and Transparency files (http://db.comparedatabase.org/documentation).

Supplementary Table S5. Quantitative outcomes of the COMPARE 2021 screening and evaluation process

Source	Reviewed "candidate allergens"	Reviewed articles	Approved allergens
AllergenOnline, IUIS, Literature search	3	3	3
AllergenOnline, UniProt	2	1	0
AllergenOnline, Uniprot, IUIS	1	1	1
NCBI Protein, Literature search	8	1	0
NCBI Protein, UniProt, Literature search	5	1	0
IUIS	2	2	0
IUIS, Literature search	3	3	3
Literature search	126	36	66
Other	21	2	21
UniProt, IUIS	1	1	1
UniProt	37	12	8
**Total**	209	63	**103**

The table describes the number of candidate allergens identified through the various sources and the final number of allergens accepted in COMPARE 2021, for each category, after peer review panel review.

Abbreviations: COMPARE, COMprehensive Protein Allergen REsource; IUIS, World Health Organization/International Union of Immunological Societies Allergen Nomenclature Database.

Appendix-1: Direct literature search strategy (methods supplement)

The complementary targeted literature search strategy applied to support the build of COMPARE 2018 and 2019 was based on the following key terms, designed according to the project scope:

(("Immunoglobulin E" or IgE or antibod* or protein* or peptid* or polypeptide*) near/5 (Allergen* or allergenicit* or allergenic or hypersensitive)) *AND* (detect* or evaluat* or characteri* or identif* or "cross-react*")

Searches were run through the Web of Science Core Collection (https://clarivate.com/products/web-of-science) including BIOSIS, CAB, and Medline for articles published in 2016 and 2017 (until August 2017) for COMPARE 2018; for COMPARE 2019, the following databases were screened in addition to the original databases screened: PubMed, ToxNet, Biological Abstracts, BIOSIS Previews, Embase, for articles published between August 1, 2017 and May 15, 2018 for COMPARE 2019. For COMPARE 2020 and 2021, Scopus was screened in addition to the databases mentioned above.

Appendix-2: 2017 COMPARE database build process and quantitative outcomes of the screening process. (information supplement to Figure 2)

The filtered results identified 55,641 protein sequences. Secondary searches of the NCBI NR protein database using specific keywords (e.g., "profilin", "tropomyosin", etc.; see Supplementary Table S1) were also performed. The search was done by replacing "allerg*" in the above-mentioned Boolean search with each of the defined specific keywords, resulting in the identification of 15,704 sequences. All the candidate sequences derived from the above search were downloaded in GenPept format and subjected to the keyword-based filtering process.

After initial filtering, 568 of the 55,641 entries from the "allerg*" search were retained. From the secondary searches that retrieved 15,704 sequences, filtering using the same steps resulted in 65 sequences. Final filtering removed those that lacked references or were irrelevant to the potential identification of a new allergen sequence (e.g., extraction/detection methods of allergens; characterization/quantification of allergens; structural studies; patient-related/treatment methods). Redundant (identical) sequences were removed as well, resulting in 251 candidate sequences eligible for PRP review. The PRP approved a total of 14 new sequences to be added to the foundational 1956 sequences from AllergenOnline v.16, resulting in 1970 total sequences in COMPARE 2017.
